# Enhancing Approaches to Detect Papilloma-Associated Hyperostosis Using a Few-Shot Transfer Learning Framework in Extremely Scarce Radiological Datasets

**DOI:** 10.3390/diagnostics16020311

**Published:** 2026-01-18

**Authors:** Pham Huu Duy, Nguyen Minh Trieu, Nguyen Truong Thinh

**Affiliations:** Institute of Intelligent and Interactive Technologies, University of Economics Ho Chi Minh City—UEH, Ho Chi Minh City 700000, Vietnamtrieunm@ueh.edu.vn (N.M.T.)

**Keywords:** PAH detection, transfer learning, papilloma-associated hyperostosis, *n*-small data, Vietnamese case study

## Abstract

**Background/Objectives:** The application of deep learning models for rare diseases faces significant difficulties due to severe data scarcity. The detection of focal hyperostosis (PAH) is a crucial radiological sign for the surgical planning of sinonasal inverted papilloma, yet data is often limited. This study introduces and validates a robust methodological framework for building clinically meaningful deep learning models under extremely limited data conditions (*n* = 20). **Methods:** We propose a few-shot learning framework based on the nnU-Net architecture, which integrates an in-domain transfer learning strategy (fine-tuning a pre-trained skull segmentation model) to address data scarcity. To further enhance robustness, a specialized data augmentation technique called “window shifting” is introduced to simulate inter-scanner variability. The entire framework was evaluated using a rigorous 5-fold cross-validation strategy. **Results:** Our proposed framework achieved a stable mean Dice Similarity Coefficient (DSC) of 0.48 ± 0.06. This performance significantly outperformed a baseline model trained from scratch, which failed to converge and yielded a clinically insignificant mean DSC of 0.09 ± 0.02. **Conclusions:** The analysis demonstrates that this methodological approach effectively overcomes instability and overfitting, generating reproducible and valuable predictions suitable for rare data types where large-scale data collection is not feasible.

## 1. Introduction

Sinonasal inverted papilloma (SIP) is a benign epithelial neoplasm characterized by local aggressiveness, a high risk of recurrence of about 13 to 30%, and a significant potential for malignant transformation [1,2]. Effective recurrence control is critically dependent on the complete surgical resection of the tumor’s attachment site. On computed tomography (CT), papilloma-associated hyperostosis (PAH) presents as focal bone thickening at the tumor’s origin, serving as a crucial imaging marker for this site, with a reported diagnostic accuracy of approximately 89% [2]. However, its recognition is challenging, requiring expert differentiation from non-pathological inflammatory osteitis [3]. This clinical challenge is compounded by the disease’s rarity, as SIP accounts for only 0.4–4.7% of all sinonasal tumors. This rarity creates a clear and significant clinical need for an automated, reliable artificial intelligence model to assist in localizing the attachment site [4,5]. Efforts to develop artificial intelligence algorithms aim to diagnose diseases in conditions where there is a shortage of expert diagnoses [6,7]. The primary barrier to applying deep learning is severe data scarcity. In this study, a dataset of 20 patients (*n* = 20) was used to train the model; however, the risks of overfitting and poor generalization are significantly high. This is the biggest challenge, so a processing algorithm for scarce data is needed. A recent attempt by McKee et al. [8] applied the state-of-the-art nnU-Net framework on a considerably larger dataset (*n* = 58). Their model achieved a mean Dice similarity coefficient (DSC) of only approximately 0.34, which is insufficient for clinical use.

A critical review of other modern AI paradigms reveals they are equally unsuitable for *n*-small problems. Although few-shot learning (FSL) has emerged as a promising strategy for handling scarce data, its application to dense 3D segmentation remains computationally unstable compared to 2D tasks. While Transformer-based architectures (e.g., Swin UNETR) have shown promise in medical imaging by capturing global context [9,10], they typically lack the strong inductive biases, such as translation invariance and locality, that CNNs possess [11]. Consequently, recent benchmarks indicate that without sufficient training data to learn these properties, Transformers can underperform for well-configured CNNs in extremely low-data regimes [12,13]. Similarly, while Self-Supervised Learning (SSL) is powerful, its efficacy often relies on large-scale unlabeled pre-training, which poses challenges when the entire available cohort is extremely small [14,15]. Similarly, Generative Data Augmentation using GANs or Diffusion Models is a dead end. These models are notoriously unstable to train on small datasets and often suffer from mode collapse, producing low-fidelity and blurry images that fail to capture the subtle pathological details of PAH, thus acting as noise that harms rather than helps performance [16]. Finally, the most common strategy, out-of-domain transfer learning, is semantically irrelevant, as features for natural images do not map to subtle bone-texture analysis on CT.

This study develops and validates a robust, composite deep learning framework specifically designed to overcome these challenges regarding the dataset and accuracy of the diagnostic process. This strategic approach combines three key components to solve these identified gaps. The nnU-Net architecture is applied as a powerful and self-configuring baseline. Then, the train-from-scratch failure is solved by employing in-domain transfer learning, initializing weights from a pre-trained skull segmentation model. The limitation of standard data augmentation, which typically focuses on geometric transformations but ignores radiometric variations, is solved. A specialized “Window Shifting” technique that simulates the variability in Hounsfield Unit (HU) calibration often observed between different CT scanners is introduced. By perturbing intensity values, this method compels the model to learn robust morphological descriptors of hyperostosis rather than relying on unstable absolute intensity thresholds, thereby enhancing clinical generalizability. The results demonstrate that this synergistic framework can significantly outperform a baseline model trained from scratch, thus providing a viable pathway for building a reliable AI model with small dataset clinical scenarios. The implications of this study offer a path to guide more precise surgical resections and reduce healthcare disparities to support the diagnosis of tumor location.

This paper is organized as follows. The Materials and Methods are presented in the next section, which presents the dataset and annotation, and the proposed segmentation framework, which is the key and main contribution. The experimental design and statistical evaluation are proposed in the next section. Then, the results and discussions are presented in the corresponding section. Finally, the conclusions of this paper are presented.

## 2. Materials and Methods

### 2.1. Dataset and Annotation

**Figure 1:**
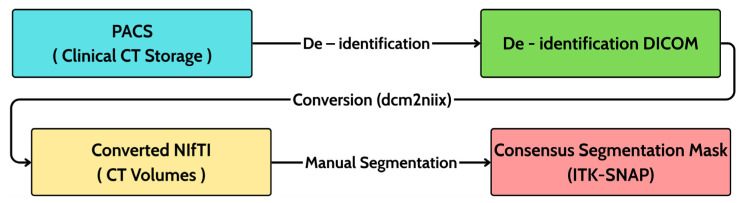
Data processing workflow.

This retrospective study analyzed 20 patients with histopathology-confirmed sinonasal inverted papilloma (SIP). Data were assembled in collaboration with otolaryngology and radiology teams; only de-identified images were retained for research, and all handling of patient information adhered to ethical principles. The criteria are biopsy-proven SIP and availability of a preoperative non-contrast paranasal sinus CT. Exclusion criteria comprised any prior sinonasal surgery, prior radiation to the sinonasal region, or severe CT artifacts that could preclude reliable assessment. For each subject, the imaging consisted of a 3D axial helical CT volume acquired in routine clinical practice without the use of intravenous contrast. To capture real-world variability, data were collected using multiple scanner platforms. Despite the limited sample size (*n* = 20), the cohort was carefully curated by the radiological team to encompass a representative spectrum of PAH morphologies, ranging from subtle focal thickening to extensive neo-osteogenesis, while excluding cases with severe artifacts. Typical in-plane resolution was 512 × 512 in native DICOM, and the number of slices and voxel spacing (Δ*x*, Δ*y*, Δ*z*) varied by scanner protocol. After retrieval from the institutional PACS, DICOM headers were de-identified, and volumes were converted to NIfTI-1 using dcm2niix while preserving affine geometry and orientation. Each case was stored as a paired set comprising the CT volume (NIfTI, floating-point, HU) and a binary label mask (NIfTI, unsigned 8-bit) co-registered on the same grid and affine. A versioned index recorded anonymized IDs, file paths, voxel spacing, slice counts, and quality flags to ensure traceability. Curation followed a three-stage workflow as initial screening against eligibility criteria; acquisition–de-identification–conversion, two-tier quality control (QC) comprising automated integrity and subsequent radiologist visual review to confirm adequate sinonasal coverage and to exclude prohibitive artifacts that are shown in Figure 1.

**Figure 2:**
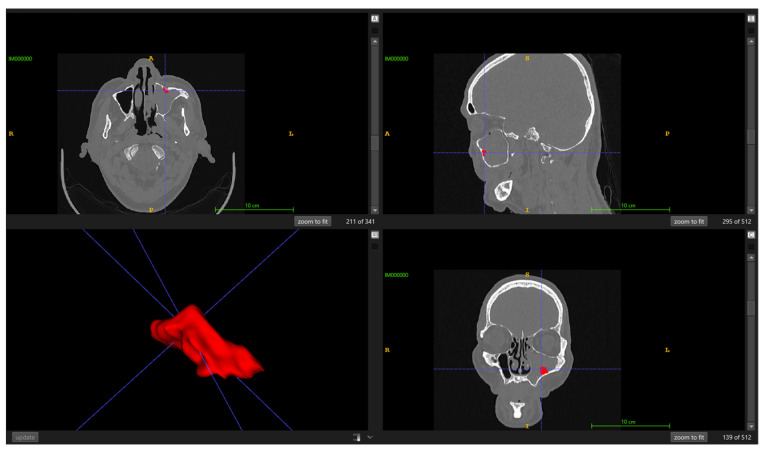
Illustration of the ground truth annotation process using ITK-SNAP ver 3.8.0. (1) The letters denote standard anatomical orientations: A (Anterior), P (Posterior), R (Right), L (Left), S (Superior), and I (Inferior). (2) The blue crosshair lines represent the navigation cursor, indicating the intersection of the axial, sagittal, and coronal planes for 3D triangulation. (3) The red overlay corresponds to the consensus binary mask of the Papilloma-Associated Hyperostosis (PAH), manually delineated by radiologists.

The target is Papilloma-Associated Hyperostosis (PAH), i.e., focal hyperostosis at the presumed tumor attachment site, and explicitly differentiated from diffuse osteitis of chronic rhinosinusitis. Ground truth was established via consensus manual segmentation in ITK-SNAP version 3.8.0 by two board-certified radiologists with over 10 years of experience. To ensure standardized visualization, a constant bone window with the window level set to 500 HU and a window width of 2000 HU was applied. Before formal annotation, the readers conducted a calibration session to harmonize boundary rules (transitional bone, thin/irregular plates, partial-volume effects). For each case, a single consensus mask was produced according to the imaging criteria of Lee DK et al. [2] to ensure reliability and minimize inter-observer variability, where focal bony thickening/neo-osteogenesis localized to the sinus wall indicates the SIP attachment site and must be distinguished from non-focal inflammatory change. Final masks were saved as binary NIfTI volumes co-registered to their corresponding CT, as illustrated in Figure 2.

### 2.2. Data Preprocessing and Augmentation

#### 2.2.1. Preprocessing

Before training, a harmonization step is performed to ensure spatial correspondence between images and labels. Each segmentation mask is resampled to match the spatial grid of its corresponding CT image, including origin, spacing, and orientation, using nearest-neighbor interpolation to preserve binary label integrity. All cases were then processed using the fully automated preprocessing pipeline of nnU-Net. Based on an analysis of dataset properties, nnU-Net automatically configured the core preprocessing steps. Specifically, all volumes were resampled to a target median voxel spacing of 0.6 × 0.46875 × 0.46875 mm. Intensities are subsequently clipped to a Hounsfield Unit (HU) window suitable for bone structures and normalized via z-score standardization, thereby ensuring consistency across patients and scanners.

#### 2.2.2. Input Formulation and Augmentation

Before entering the backbone network, all CT volumes were preprocessed and augmented following the standard nnU-Net pipeline [17], with additional modifications introduced to enhance robustness under extremely small-sample conditions. All CT volumes were first resampled to an isotropic voxel spacing of 0.6 × 0.46875 × 0.46875 mm^3^ and clipped to a bone-specific Hounsfield Unit (HU) window [−1000, 3000], suppressing non-relevant soft-tissue signals. Intensity values were then standardized by z-score normalization as in Equation (1):

(1) $$I^{\prime }=\frac{I-\mu }{\sigma }$$

where *I* denotes the voxel intensity; *μ*, *σ* represent the mean and standard deviation within the corresponding non-zero voxel distribution.

During training, on-the-fly data augmentation is applied to mitigate overfitting and improve generalization. The default nnU-Net transformations included random rotations (±15°), scaling (0.9–1.1), elastic deformations, and mirroring along random anatomical planes. In addition to these built-in augmentations, we introduced a custom window-shifting augmentation to simulate inter-scanner variability in bone-window calibration. After normalization [18,19], the voxel intensities were globally perturbed with a probability *p* = 0.3 as in Equation (2):

(2) $$I^{\prime \prime }=I^{\prime }+\alpha ,\alpha \sim U(-0.1,0.1)$$

where *α* is a uniformly distributed random shift.

This technique differs fundamentally from conventional density-based enhancements such as random gamma correction or brightness scaling, which typically alter the dynamic range or distribution shape. Instead, Window Shifting applies a linear translational offset to simulate the systematic calibration bias in Hounsfield Units often observed between different scanner manufacturers. This encourages the network to learn morphological rather than absolute intensity cues, thereby improving cross-scanner robustness.

Training is conducted on randomly sampled 3D patches of size 80 × 192 × 160 voxels, ensuring a balanced sampling of lesion-containing and background regions [17]. During inference, overlapping patch predictions were aggregated by weighted averaging and thresholded at 0.5 to reconstruct the final binary segmentation mask. This combination of standardized preprocessing and both default and custom augmentations effectively reduced overfitting and enabled the proposed few-shot framework to learn stable, generalizable representations from only 20 subjects.

### 2.3. Proposed Segmentation Framework

#### 2.3.1. Overall Design and Rationale

The automated segmentation of papilloma-associated hyperostosis (PAH) on computed tomography (CT) scans poses a formidable challenge because the target lesions are typically small, subtle, and located adjacent to complex bony interfaces, which renders them difficult to distinguish from normal anatomical variations or chronic inflammatory changes [1]. Previous work attempting to tackle this task with the well-established nnU-Net framework on a dataset of 58 patients achieved a mean Dice similarity coefficient (DSC) of approximately 0.34, thereby highlighting the severe barrier imposed by limited sample size [8]. In our setting, this difficulty is even more acute, as only 20 patient scans are available. Attempting to train a high-capacity neural network from scratch under such constraints would be expected to result in severe overfitting and instability, ultimately leading to poor generalization performance on unseen data and unreliable clinical predictions [20].

To mitigate these issues, a principled framework is designed that integrates the standardized nnU-Net v2 pipeline, specifically its 3d_fullres configuration, with an in-domain transfer learning strategy that leverages prior knowledge from a closely related segmentation task. However, recent years have witnessed the emergence of Transformer-based architectures such as UNETR and Swin UNETR, which are theoretically capable of capturing long-range contextual dependencies [21]. The decision to rely on a convolutional neural network (CNN) based U-Net is not simply a matter of tradition but rather a deliberate, evidence-based choice supported by both methodological studies and the specific clinical nature of PAH. When compared under identical conditions within the nnU-Net pipeline, a well-configured CNN-based U-Net remained highly competitive and frequently outperformed Transformer-based alternatives across diverse datasets.

Furthermore, the biological and radiological characteristics of PAH itself favor a CNN-based solution. The lesion is a localized phenomenon, expressed as fine-grained textural and morphological changes confined to the bony sinus wall. Such localized features are precisely the type of patterns that convolutional kernels are optimized to detect, while the primary strength of Transformers, with modeling global contextual relationships, is of secondary importance in this application [21]. In addition, the effectiveness of combining nnU-Net with in-domain transfer learning has been demonstrated in related small-data scenarios. For example, Bareja et al. [22] successfully adapted a model pre-trained on adult gliomas to segment pediatric medulloblastomas, achieving robust performance across multiple institutions despite limited sample sizes.

Building upon these insights, our solution involves initializing an optimized 3D U-Net with weights derived from a skull segmentation model available in the PYCAD Model Zoo and subsequently fine-tuning it within the nnU-Net v2 framework. This approach is grounded in rigorous empirical evidence, exploits the proven strengths of CNN-based architectures for localized feature extraction, and directly addresses the fundamental challenge of extreme data scarcity by harnessing in-domain transfer learning.

#### 2.3.2. Model Architecture

Building upon the preprocessed dataset described in Section 2.1, the proposed framework employs the three-dimensional U-Net architecture exactly as implemented in the standardized 3d_fullres configuration of nnU-Net. The following description details the key components and design principles of this specific, well-established framework, which our study adopts without modification. This architectural choice is deliberate to design the symmetric encoder–decoder of U-Net, which is uniquely suited to capturing features across multiple spatial scales. The encoder progressively down samples the input to extract high-level contextual information, while the decoder reconstructs spatial resolution. Crucially, skip connections relay high-resolution feature maps from the encoder to their corresponding decoder layers, enabling precise localization of small, subtle targets such as Papilloma-Associated Hyperostosis (PAH) while maintaining awareness of the broader anatomical context. The network follows a six-stage hierarchy, with encoder feature channels increasing to learn abstract representations. Specifically, the encoder utilizes 3 × 3 × 3 convolutional kernels with a starting feature map size of 32 channels, which doubles at each subsequent down-sampling stage (32, 64, 128, 256, 320, 320). The model processes a 3D input patch of size 80 × 192 × 160 voxels, producing a binary segmentation mask of identical dimensions. At each convolutional layer *l*, the transformation of a 3-D input patch $x\in {\mathbb{R}}^{C_{in}\times D\times H\times W}$ is defined as (3):

(3) $$y_{k}^{\left(l\right)}=\phi \left(\mathrm{IN}\left(\sum_{c=1}^{C_{\mathrm{in}}}W_{k,c}^{\left(l\right)}*x_{c}+b_{k}^{\left(l\right)}\right)\right)$$

where $W_{k,c}^{\left(l\right)}\in {\mathbb{R}}^{K\times K\times K}$ denotes the 3-D convolution kernel (K=3), ∗ represents the convolution operator, IN (⋅) is Instance Normalization, $b_{k}^{\left(l\right)}$ is the bias term, and *ϕ* (⋅) is the Leaky ReLU activation function with a negative-slope coefficient *α* = 0.01 as (4).

(4) $$\phi (x)=\{\begin{array}{cc} x, & x\geq 0\\ \alpha x, & x<0 \end{array}$$

The total number of parameters per layer is $K^{3}C_{in}C_{out}+C_{out}$. Each encoder block contains two consecutive 3 × 3 × 3 convolutions, each followed by Instance Normalization and Leaky ReLU activation, expressed as (5).

(5) $$f^{\left(l\right)}=\phi \left(\mathrm{IN}\left({\mathrm{Conv}}_{3^{3}}\left(\phi \left(\mathrm{IN}\left({\mathrm{Conv}}_{3^{3}}\left(f^{\left(l-1\right)}\right)\right)\right)\right)\right)\right)$$

Between stages, spatial resolution is reduced using strided convolution with a stride of 2, which is a parametric down-sampling operation that replaces max pooling as in Equation (6).

(6) $$f_{↓}^{\left(l\right)}=\mathrm{Con}v_{3^{3}}^{s=2}(f^{\left(l\right)})$$

This approach allows the network to learn adaptive feature compression, which is beneficial for capturing subtle osseous texture variations in CT data. Symmetrically, the decoder pathway progressively up-samples feature maps to reconstruct the full spatial resolution. This is achieved through transposed convolutions with a kernel with a stride of 2, which is shown in (7).

(7) $$g_{↑}^{\left(l\right)}={\mathrm{ConvTranspose}}_{2^{3}}^{s=2}\left(g^{\left(l+1\right)}\right)$$

The output of this up-sampling step is then concatenated with the corresponding feature map from the encoder via a skip connection (8).

(8) $${\overset{˜}{g}}^{\left(l\right)}=\mathrm{Concat}\left(g_{↑}^{\left(l\right)},f^{\left(l\right)}\right)$$

Each concatenated tensor subsequently passes through a decoder block identical in structure to the encoder block, a process that refines spatial details lost during down-sampling. These skip connections are crucial for preserving fine-grained boundaries, such as cortical bone interfaces and localized PAH. The output head of the network projects the final decoder feature map to the class space using a 1 × 1 × 1 convolution followed by a voxel-wise softmax function (9).

(9) $$p=\mathrm{Softmax}\left({\mathrm{Conv}}_{1^{3}}\left({\overset{˜}{g}}^{\left(0\right)}\right)\right)$$

This produces a dense probability map $\rho \in {\left[0,1\right]}^{C\times D\times H\times W}$ for *C* = 2 classes. In the nnU-Net v2 paradigm, auxiliary prediction heads are attached to intermediate decoder stages to enable deep supervision. During training, these auxiliary outputs, denoted as $p^{\left(s\right)}$ at scale s, are upsampled to the native resolution and contribute to the total loss, thereby improving gradient propagation across multi-scale representations. An important aspect of this architecture is its receptive field (RF). For a kernel size K = 3 and stage-wise strides *s_j_ ∈ (1,2)*. The effective RF after the 1st stage follows the relation (10).

(10) $$R_{l}=R_{l-1}+\left(K-1\right)\prod_{j=1}^{l-1}s_{j}$$

**Figure 3:**
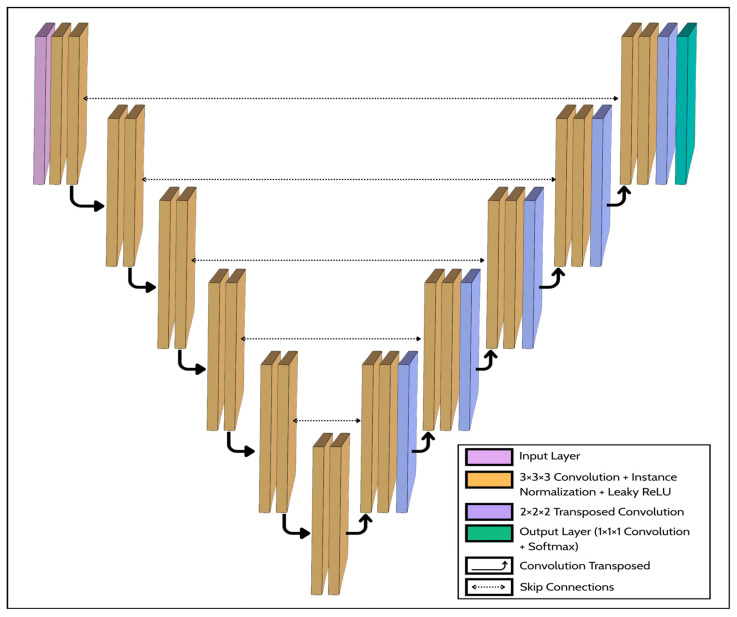
Schematic of the proposed 3D U-Net architecture for Papilloma-Associated Hyperostosis (PAH) segmentation.

After five down-sampling operations, the theoretical RF spans the entire 80 × 192 × 160 voxel Three-dimensional patch, enabling the model to integrate long-range sinus-bone context while maintaining high-resolution spatial fidelity through the skip pathways. The design rationale for this architecture is based on several key principles. 3-D kernels are employed because the dataset has nearly isotropic voxel spacing, allowing the network to exploit inter-slice continuity critical for sinonasal morphology. This stands in contrast to conventional 2D U-Net approaches, which process volumes slice-by-slice and consequently fail to capture the volumetric contextual information essential for defining the 3D structure of PAH lesions. Instance Normalization stabilizes feature scaling under small-batch training and reduces dependency on absolute HU values. Strided convolution for down-sampling and transposed convolution for up-sampling preserve learnable spatial mappings, avoiding artifacts from pooling/unpooling. Leaky ReLU ensures non-zero gradients for negative activations, mitigating dead-filter effects in low-contrast osseous regions. Finally, the architecture excludes dropout layers by default to prevent information loss in extremely small-sample settings. A schematic of the proposed 3D U-Net architecture for Papilloma-Associated Hyperostosis segmentation is shown in Figure 3.

Overall, this design is grounded in both empirical and clinical considerations. The use of 3D convolutional kernels leverages the near-isotropic voxel spacing, enabling the model to exploit inter-slice continuity essential for sinonasal morphology. Instance Normalization stabilizes training under small-batch conditions and reduces dependency on absolute HU values. Strided and transposed convolutions preserve learnable spatial mappings, avoiding the artifacts introduced by pooling and unpooling. The Leaky ReLU activation ensures non-zero gradients even in low-contrast osseous regions, minimizing dead-filter effects. Finally, the architecture omits dropout layers to prevent information loss in the small-sample regime. Collectively, these design choices enable the 3D U-Net backbone to achieve a robust balance between spatial precision, contextual awareness, and data efficiency in segmenting Papilloma-Associated Hyperostosis.

#### 2.3.3. Transfer Learning Strategy

Training a deep neural network with millions of parameters from scratch on an extremely small dataset (*n* = 20) is fraught with challenges. With random weight initialization, the model would likely fail to learn meaningful representations and would be highly susceptible to overfitting, where it memorizes the training data instead of learning generalizable features, resulting in poor performance on unseen cases and unreliable validation metrics [9]. To mitigate these risks, a transfer learning strategy is adopted. The fundamental benefit of this approach is that it leverages knowledge from a related, large-scale task to provide the model with a strong initial foundation [2]. Instead of starting from a random state, the model’s weights are initialized from a network that has already been trained to understand relevant low-level features. This provides a much better starting point for optimization, accelerates convergence, and significantly improves the model’s ability to learn from limited data. In this study, an in-domain transfer learning approach is employed. This choice is highly strategic because PAH manifests as localized thickening and neo-osteogenesis at bone interfaces, a model already proficient in segmenting complex cranial bone structures, possesses rich shape and texture priors that are far more relevant to this task than features learned from natural image datasets like ImageNet. Then, fine-tuning all layers of this pre-trained model on 20 subjects is performed, allowing the network to adapt its learned knowledge to the specific task of identifying PAH. The fine-tuning process leveraged the robust optimization framework of the nnU-Net model. The optimization settings were conducted using the default nnU-Net v2 optimization settings. Crucially, the fine-tuning utilized the full data augmentation pipeline described in Section 2.2.2, which includes both the standard nnU-Net transformations and our custom window-shifting augmentation designed to enhance cross-scanner robustness. A patient-level 5-fold cross-validation was employed to prevent data leakage and provide a robust performance estimate. The transfer learning process can be expressed as a domain mapping as (11).

(11) $$F_{\mathrm{pre}}:A\to B$$

where *A* represents the skull segmentation model pretrained on large-scale cranial CT data, and *B* represents the PAH segmentation.

The pretrained network $F_{\mathrm{pre}}$ provides initial parameters *θ_A_*, which are fine-tuned to *θ_B_* through gradient updates on the small PAH dataset (12).

(12) $$\theta_{B}=\theta_{A}+\Delta \theta$$

where Δθ denotes the domain adaptation achieved during few-shot fine-tuning. This mapping efficiently transfers anatomical priors, accelerating convergence and improving generalization under extreme data scarcity.

#### 2.3.4. Output Layer and Fully Connected Equivalent

Unlike classification networks ending with a fully connected layer, the 3D U-Net’s final convolution acts as its fully connected equivalent, performing dense voxel-level classification [6]. For each voxel *v*, *p_v_* is calculated as in Equation (13).

(13) $$p_{v}=\mathrm{softmax}(W\times F_{v}+b)$$

where *F_v_* is the final feature vector, *W* and b are learnable parameters.

**Figure 4:**
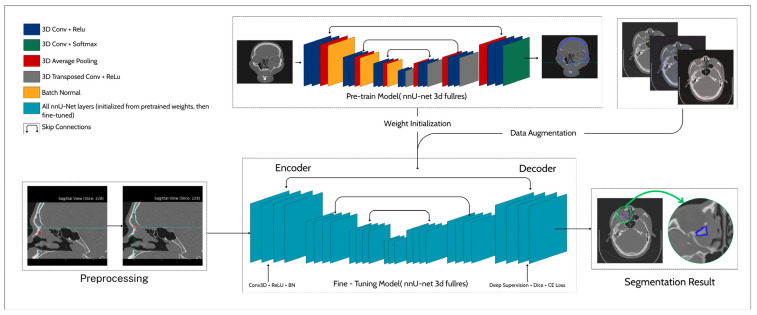
Proposed pipeline for hyperostosis segmentation.

This produces a 3D probability map *p(x*,*y*,*z),* thresholded at 0.5 to yield the binary segmentation mask. The output thus represents a continuous volumetric probability field, preserving spatial structure and enabling detailed delineation of hyperostotic regions. This is a more informative outcome than a single global prediction. Clinically, these voxel-wise outputs highlight bony attachment zones of sinonasal inverted papilloma, providing quantitative and visual guidance for preoperative planning. The framework integrates preprocessing, transfer learning from a pre-trained skull model, and data augmentation with window shifting into a fine-tuned 3D U-Net with deep supervision, optimized by Dice combined with cross-entropy loss, yielding robust segmentation results on a scarce SIP CT dataset (*n* = 20). The framework begins with a preprocessing stage in which sinonasal CT volumes are de-identified, resampled, and intensity-normalized to ensure spatial and radiometric consistency. The pre-trained nnU-Net model is originally trained for skull segmentation and provides the encoder–decoder weight initialization used for in-domain transfer learning. During fine-tuning, all network layers are updated under a composite loss function combining Soft Dice and Cross-Entropy terms, while deep supervision is applied through auxiliary decoder outputs to stabilize gradient propagation. The pipeline also integrates a specialized window-shifting augmentation, which introduces controlled intensity perturbations to improve cross-scanner generalization. The final fine-tuned model produces voxel-wise probability maps highlighting focal hyperostosis regions corresponding to tumor attachment sites on sinonasal CT slices. This integrated strategy combines transfer learning and targeting augmentation to enable robust segmentation performance even under extreme data scarcity (*n* = 20). Figure 4 shows the proposed pipeline for hyperostosis segmentation.

### 2.4. Training and Optimization

#### 2.4.1. Loss Function

The model is optimized using the standard composite loss function based on the nnU-Net model. This protocol combines the soft dice loss and the voxel-wise Cross-Entropy (CE) loss, a formulation designed to balance region-level overlap accuracy with local voxel-wise classification stability. The total loss is expressed as (14):

(14) $$L_{\mathrm{total}}=\lambda_{\mathrm{Dice}}L_{\mathrm{Dice}}+\lambda_{\mathrm{CE}}L_{\mathrm{CE}}$$

where $\lambda_{\mathrm{Dice}}$ and $\lambda_{\mathrm{CE}\;}$ denote the weighting coefficients.

The Soft Dice loss directly optimizes spatial overlap between prediction and ground truth, effectively mitigating the severe class imbalance inherent in PAH segmentation. It is defined as (15):

(15) $$L_{\mathrm{Dice}}=1-\frac{2\sum_{i=1}^{N}p_{i}g_{i}+\epsilon }{\sum_{i=1}^{N}(p_{i}^{2}+g_{i}^{2})+\epsilon }$$

where $p_{i}$ and $g_{i}$ represent the predicted probability and ground-truth label of a voxel i, respectively, *N* is the total number of voxels, and $\epsilon =10^{-6}$ ensures numerical stability. Complementarily, the Cross-Entropy loss provides smooth per-voxel gradient feedback, stabilizing the training process and improving convergence. It is formulated as (16).

(16) $$L_{\mathrm{CE}}=-\frac{1}{N}\sum_{i=1}^{N}\left[g_{i}\log(p_{i})+(1-g_{i})\log(1-p_{i})\right]$$

This combined formulation enhances both global shape agreement and local boundary precision, leading to more reliable segmentation results. Training was performed on 3D patches of 80 × 192 × 160 voxels with a batch size of 2, allowing efficient GPU utilization while preserving volumetric context. To further stabilize gradient flow, the framework employs deep supervision by attaching auxiliary outputs to intermediate decoder stages. The multi-scale loss is computed as (17):

(17) $$L_{\mathrm{DS}}=\sum_{s=1}^{S}\alpha_{s}L_{\mathrm{total}}^{\left(s\right)}$$

where S denotes the number of supervised scales and *α_s_* are decreasing weights for deeper layers.

The entire fine-tuning process followed the nnU-Net optimization protocol, using stochastic gradient descent (SGD) with Nesterov momentum (μ = 0.99) and a polynomial learning rate decay defined as Equation (18):

(18) $$\eta_{t}=\eta_{0}{\left(1-\frac{t}{T_{\max}}\right)}^{0.9}$$

where $\eta_{t}$ is the learning rate at iteration t, $\eta_{0}$ is the initial learning rate, and $T_{\max}$ represents the total number of training iterations.

#### 2.4.2. Optimization Setup

The training, optimization, and inference process strictly followed the standardized protocol of nnU-Net with the transfer learning for weight initialization. Given the large memory footprint of volumetric CT data and the limited number of annotated cases, training was performed on 3D patches of modified size. This configuration was selected as an empirically verified trade-off between GPU memory efficiency and contextual coverage, ensuring that each patch fully encapsulated the sinonasal anatomy relevant to Papilloma-Associated Hyperostosis. A stochastic gradient descent (SGD) with Nesterov momentum to accelerate convergence and stabilize training dynamics is used, with the update rule at iteration t can be expressed as (19):

(19) $$\begin{array}{cc} v_{t+1} & =\mu v_{t}-\eta_{t}\nabla_{\theta }L(\theta_{t}+\mu v_{t})\\ \theta_{t+1} & =\theta_{t}+v_{t+1} \end{array}$$

where *θ_t_* denotes the trainable parameters at iteration t, $v_{t}$ is the velocity term, μ=0.99 represents the Nesterov momentum coefficient, and *η_t_* is the learning rate. And the initial learning rate is chosen as (20) to maintain smooth convergence:

(20) $$\eta_{t}=\eta_{0}{\left(1-\frac{t}{T}\right)}^{p}$$

where T denotes the total number of training iterations and p=0.9 controls the rate of decay.

To ensure stable gradient propagation across the deep architecture, deep supervision was employed by attaching auxiliary segmentation heads to intermediate decoder stages. Each auxiliary output Y^(s) at scale s contributed to the overall multi-scale loss function as (21):

(21) Ltotal=∑s=1SwsL(Y^(s),Y)

where Y is the ground-truth mask, L denotes the composite Dice–Cross-Entropy loss, and $w_{s}$ are scale-specific weighting coefficients that decrease exponentially with layer depth ($w_{s}=1/2^{s-1}$).

This hierarchical supervision framework ensures that both shallow and deep layers receive informative gradient signals, mitigating vanishing gradients and promoting balanced feature learning across multiple resolutions. Weight initialization followed the transfer learning paradigm to make sure that all layers were fine-tuned end-to-end without freezing, allowing full adaptation to the PAH domain while retaining low-level structural priors related to craniofacial bone morphology. Regularization is implicitly achieved through a combination of data augmentation, deep supervision, and transfer learning, with no dropout layers applied, consistent with nnU-Net’s design for small-batch stability. Gradient clipping at a maximum L2-norm of 12 was employed to prevent exploding gradients. Each training run was repeated across five cross-validation folds, ensuring independence between training and validation subsets to eliminate data leakage. The model with the best mean Dice score on the validation set of each fold was retained for inference. During testing, overlapping patch predictions are aggregated via Gaussian-weighted averaging, and a probability threshold is applied to produce the final binary segmentation mask. This approach yielded smooth probability transitions between patches and minimized boundary artifacts.

#### 2.4.3. Validation Strategy

Given the extremely limited dataset size (*n* = 20), the design of the validation protocol was critical to obtain statistically reliable and unbiased estimates of model performance. Following best practices in medical research [23], a five-fold cross-validation scheme is adopted to maximize data utilization while maintaining strict separation between training and validation samples. The dataset was randomly partitioned into five non-overlapping folds at the patient level, ensuring that no slices or volumes from the same subject appeared in both training and validation subsets, thereby eliminating the risk of data leakage [24]. This process was repeated five times so that every patient contributed exactly once to the validation set. To address the potential instability inherent in small validation splits (4 cases per fold), we implemented a stratified sampling strategy based on lesion volume. This ensures that each fold contains a representative distribution of both subtle (hard-to-segment) and extensive (easier-to-segment) PAH cases. By balancing the difficulty across folds, we minimized the risk of having a specific fold dominated by outliers, thereby stabilizing the error rate and ensuring a more reliable performance estimate. Each fold was trained independently from scratch using the fine-tuned pre-trained weights as initialization. The validation subset was not used for any form of model selection, early stopping, or hyperparameter tuning beyond the automatically configured nnU-Net pipeline, thereby preserving the integrity of the evaluation. Within each training session, the best-performing model checkpoint was selected based on the lowest validation loss and highest Dice Similarity Coefficient (DSC) observed during training. The primary evaluation metric was the Dice Similarity Coefficient (DSC), which measures the spatial overlap between the predicted segmentation (P) and the ground truth mask (G) as (22).

(22) $$\mathrm{DSC}=\frac{2|P\cap G|}{|P|+|G|}$$

To complement the quantitative analysis, qualitative visual inspection was performed for each validation case by two radiologists to assess spatial alignment between predicted PAH regions and the manually annotated ground truth. To assess the robustness of the framework, the variance across folds was explicitly analyzed. A low standard deviation in DSC (<0.06) indicated consistent performance across different validation splits, suggesting stable learning behavior and minimal dependence on specific training subsets. This multi-fold evaluation thus provides a statistically sound and generalizable estimate of the model’s true performance under conditions of extreme data scarcity. Given the extremely small sample size, the choice of validation method is critical to avoid biased performance estimates. A 5-fold cross-validation strategy is employed, which is a standard and robust method widely used in medical imaging research, including similar studies with small datasets [8,10]. This approach provides a good balance between bias and variance: the dataset was split into 5 folds, with each fold using 16 cases for training and 4 for validation. Using a validation set of 4 cases offers a more stable performance estimate during training compared to a single-case validation, reducing the impact of outliers. The final performance was calculated by averaging the Dice Similarity Coefficient (DSC) across all 5 folds. The DSC was chosen as the primary metric as it is the gold standard for assessing spatial overlap in medical segmentation tasks.

## 3. Results

All experiments are conducted on an NVIDIA RTX 4090 GPU with mixed precision (AMP) enabled for computational efficiency. The rigorous 5-fold cross-validation yielded stable and clinically relevant segmentation results. Our proposed framework, leveraging in-domain transfer learning and specialized data augmentation, achieved a mean Dice Similarity Coefficient (DSC) of 0.48 ± 0.06 across the 5 validation folds. This level of performance indicates a substantial spatial overlap between the model’s predictions and the expert-defined ground truth. A crucial component of our study was the ablation experiment comparing this approach to a baseline model. As shown in Table 1, the model trained from scratch on the same 5-fold cross-validation splits failed to converge to a meaningful solution, resulting in a poor and clinically insignificant mean DSC of 0.09 ± 0.02. Furthermore, we compared our results with existing state-of-the-art benchmarks for this specific pathology. Our proposed framework (DSC 0.48) demonstrates a substantial improvement over the standard nnU-Net implementation reported by McKee et al. [8], which achieved a DSC of only 0.34 despite utilizing a dataset nearly three times larger (*n* = 58). This indicates that standard SOTA methods struggle with the subtle features of PAH, whereas our proposed Transfer Learning and Window Shifting strategy effectively captures these nuances. This stark contrast underscores the infeasibility of standard deep learning approaches in this data-scarce regime and provides strong evidence for the necessity of our proposed methodological framework. To rigorously validate this improvement, a paired *t*-test was conducted comparing the fold-wise Dice scores of the proposed framework against the baseline. The analysis yielded a *p*-value of *p* < 0.001, confirming that the performance gain is statistically significant. Furthermore, we calculated the 95% Confidence Interval (CI) for the proposed model’s mean DSC, resulting in a range of (0.427, 0.533). This narrow interval, relative to the mean, further reinforces the stability and reliability of the reported results despite the small sample size. And the detailed performance of the proposed framework across each validation fold is presented in Table 2. The individual Dice scores, ranging from 0.39 to 0.54, demonstrate consistent performance across different data splits.

**Figure 5:**
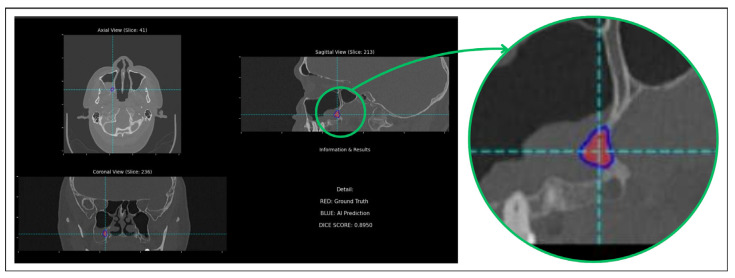
Qualitative comparison showing high segmentation accuracy between the model’s prediction (blue overlay) and the ground truth (red overlay), achieving a Dice score of 0.8950.

To better understand these quantitative results, a qualitative analysis of the segmentation outputs is performed. For this purpose, a custom software tool is developed to visualize the base CT images with overlays for both ground truth and AI-predicted segmentations. It also included features for quantitative assessment, such as real-time Dice score calculation, and functions to automatically navigate to slices with the largest segmentations or maximal prediction discrepancies, facilitating a targeted analysis of model performance. This detailed visual inspection revealed that while the mean DSC is moderate, the model is capable of achieving excellent results on a subset of the data. For instance, Figure 5 showcases a representative example of the model’s upper-bound performance (DSC = 0.8950). This case exemplifies a scenario where the radiological signs of hyperostosis are distinct, allowing the model to perform an accurate delineation. The predicted segmentation mask (blue) demonstrates a high degree of concordance with the ground truth outline (red), capturing the lesion’s morphology and extent with high precision. Presenting this successful case is intended to illustrate the model’s learned capabilities and its potential clinical utility when faced with non-ambiguous pathology.

**Figure 6:**
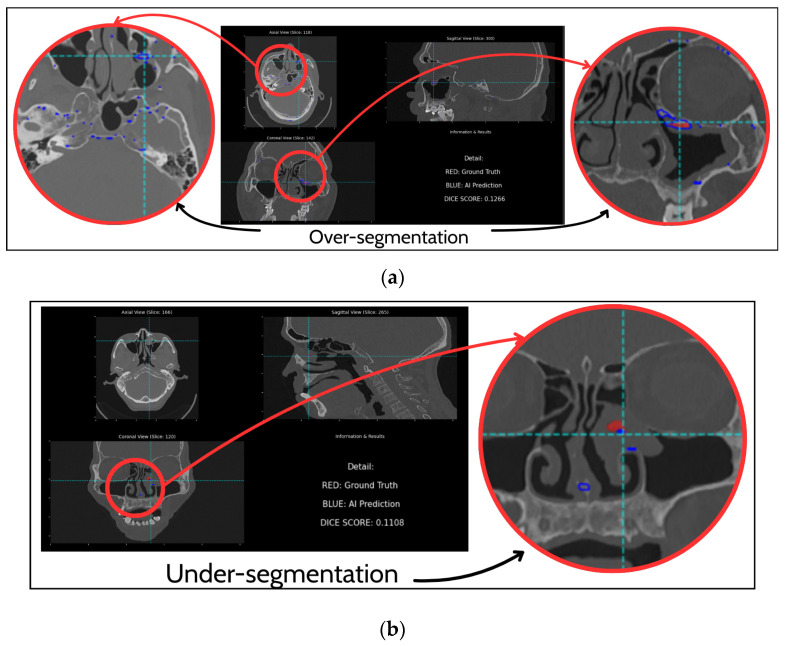
Examples of segmentation failure modes. (**a**) Over-segmentation; (**b**) under-segmentation.

A critical examination of cases with lower DSC scores revealed two primary modes of failure, as illustrated in Figure 6. In some instances, the model’s lack of specificity led to significant over-segmentation errors. A severe example is illustrated in Figure 6a, where the AI Prediction (blue) vastly overestimates the lesion’s extent. While the ground truth (red) is localized to the left ethmoid sinus and nasal septum, the model’s prediction incorrectly expands to include large areas of normal anatomy, encompassing bilateral ethmoid and sphenoid sinuses. This error, resulting in a low DSC of 0.1266, suggests that while the model attempts to identify bone thickening, its specificity is severely challenged in anatomically complex regions, where it struggles to distinguish pathological hyperostosis from benign physiological sclerosis. False negatives are predominantly observed in cases with subtle or small lesions. Figure 6b provides a clear example, where the ground truth (red) indicates a distinct lesion, but the AI prediction (blue) is almost non-existent, resulting in a very low Dice score of 0.1108. This highlights a significant limitation in the model’s sensitivity, indicating a detection threshold below which subtle lesions are completely missed.

## 4. Discussion

In this study, a methodological framework is proposed to address the critical challenge of developing deep learning models from extremely scarce medical imaging data. Our approach successfully developed a model for the automated segmentation of papilloma-associated hyperostosis, a key radiological sign for surgical planning in sinonasal inverted papilloma, with a dataset of only 20 patients. By integrating in-domain transfer learning, specialized data augmentation, and a robust cross-validation strategy, this framework achieved stable and relevant segmentation performance, specifically DSC of 0.48 ± 0.06, demonstrating a viable approach for building algorithms to support clinical diagnosis. The critical importance of the proposed strategic approach is underscored by the results of the ablation study. The baseline model, an nnU-Net trained from scratch, failed to converge to a meaningful solution, yielding a clinically insignificant mean DSC of 0.09 ± 0.02. This outcome is consistent with established principles that high-capacity neural networks are prone to severe overfitting and instability when trained on very small datasets. In stark contrast, a proposed framework produced stable and valuable segmentation results, providing strong evidence that the synergistic combination of pre-trained weight initialization and clinically informed data augmentation is essential for overcoming the challenges of extreme data scarcity.

While a mean DSC of 0.48 is generally considered moderate in large-organ segmentation, its interpretation requires nuance in the context of PAH. Mathematically, the Dice coefficient heavily penalizes boundary errors for small structures; a deviation of merely a few voxels can reduce the score significantly even when the lesion is correctly localized [25]. Clinically, the primary value of this AI model is not pixel-perfect delineation, but rather the precise localization of the tumor attachment site to guide surgical drilling. Visual inspection confirms that despite the moderate DSC, the model consistently identifies the correct focal hyperostosis region in successful cases, providing actionable confidence for preoperative planning. Regarding the comparison with McKee et al. [8], one might hypothesize that our superior performance with 14% higher despite less data. However, our cohort exhibits significant heterogeneity, acquired from multiple scanner platforms (GE, Siemens) with varying slice thicknesses. Therefore, the performance gain is driven by methodological innovations, namely the use of in-domain transfer learning, which provides stronger shape priors than training from scratch and window shifting. This consistency, achieved through the synergistic combination of in-domain transfer learning and stratified cross-validation, provides strong evidence that this methodology can generate reproducible and reliable predictions, even when facing extreme data scarcity. The implications of this work, however, extend far beyond the specific task of PAH segmentation. The data scarcity problem remains one of the most significant barriers to the application of AI in thousands of rare diseases, where collecting large-scale datasets is impractical or impossible. Our study provides a detailed and reproducible blueprint for researchers facing these challenges. It demonstrates that by strategically combining powerful, self-configuring architectures like nnU-Net with in-domain transfer learning and extensively informed data augmentation, it is possible to build valuable and reproducible AI models without relying on big data. Future research should prioritize the validation of this framework on larger, multi-center datasets to rigorously evaluate its generalizability and robustness. Such studies will be crucial in determining the real-world clinical performance of the model.

Finally, regarding the choice of methodology, we deliberately prioritized in-domain transfer learning over other few-shot or augmentation strategies proposed in the recent literature. While metric-based few-shot learning methods (e.g., MAML, Prototypical Networks) show promise for 2D images, applying them to high-dimensional 3D volumetric data remains computationally unstable and susceptible to overfitting due to the exponential increase in feature space complexity [12]. Similarly, geometric interpolation techniques like Mixup or Cutmix were excluded because they generate non-physical artifacts (e.g., “ghost” bone structures) that violate anatomical plausibility. In contrast, our “Window Shifting” approach simulates a real-world physical phenomenon, specifically radiometric calibration bias, thereby preserving the structural integrity essential for clinical interpretation.

## 5. Conclusions

In conclusion, this study proposes a methodological framework that enables the development of a model for automatic Papilloma-Associated Hyperostosis identification from extremely scarce medical imaging data. A novel algorithm proposed combining specialized transfer learning, extensive data augmentation, and a rigorous validation strategy, which not only provides a potential solution for the segmentation of hyperostosis in sinonasal inverted papilloma but, more importantly, offers a proven roadmap for the wider research community facing the ubiquitous challenge of the data scarcity problem. Future work is expected to combine this method with larger data to increase model accuracy and apply it to support clinical diagnosis.

## Figures and Tables

**Table 1:** Segmentation performance of different models.

	Baseline Model	Proposed Framework
DSC	0.09 ± 0.02	0.48 ± 0.06

**Table 2:** Proposed Framework Performance per Fold.

	Dice Score
Fold 0	0.5401
Fold 1	0.3928
Fold 2	0.5274
Fold 3	0.4339
Fold 4	0.5134

## Data Availability

The original contributions presented in this study are included in the article. Further inquiries can be directed to the corresponding author.
